# Enhancing Diagnostic Accuracy in Healthcare Through AI‐Powered Medical Image Analysis

**DOI:** 10.1002/alz70862_109999

**Published:** 2025-12-23

**Authors:** Deborah Koech

**Affiliations:** ^1^ Chagua Health, Nairobi, Nairobi Kenya

## Abstract

**Background:**

Accurate prediction of neural activity during task‐based paradigms is essential for advancing computational neuroscience and improving clinical applications. Leveraging publicly available datasets, this study developed a custom machine learning model to analyze task‐specific brain activity by aggregating functional MRI (fMRI) data into 360 regions of interest (ROIs) based on the Glasser parcellation.

**Method:**

Functional data from the Human Connectome Project (HCP), involving 100 participants, was utilized for this analysis. Participants performed seven cognitive tasks, including motor, working memory, emotion, language, social cognition, gambling, and relational reasoning, with each task collected over two acquisition runs (LR and RL) at a temporal resolution of 0.72 seconds. The data were preprocessed to extract ROI‐level information, and a custom‐built neural network was trained to predict brain activity across conditions for each task.

**Result:**

The model achieved high predictive accuracy, successfully identifying task‐specific neural activation patterns across diverse cognitive paradigms. Key insights revealed robust hemispheric symmetry during motor tasks and distinct activation profiles for relational reasoning and social cognition tasks. The use of ROI‐level aggregation provided a balance between computational efficiency and spatial resolution, enhancing the interpretability of the results.

**Conclusion:**

his work demonstrates the potential of machine learning models in analyzing publicly available neuroimaging datasets to predict and characterize task‐specific brain activity. The findings underscore the importance of data‐driven approaches in uncovering neural dynamics and pave the way for applications in cognitive neuroscience and clinical interventions.

